# Men's Understanding of and Experiences During the Postcircumcision Abstinence Period: Results From a Field Study of ShangRing Circumcision During Routine Clinical Services in Kenya and Zambia

**DOI:** 10.1097/QAI.0000000000000704

**Published:** 2016-05-24

**Authors:** Mark A. Barone, Philip S. Li, Robert Zulu, Quentin D. Awori, Kawango Agot, Stephanie Combes, Raymond O. Simba, Richard K. Lee, Catherine Hart, Jaim Jou Lai, Zude Zyambo, Marc Goldstein, Paul J. Feldblum, David C. Sokal

**Affiliations:** * EngenderHealth, New York, NY;; † Center for Male Reproductive Medicine and Surgery, Department of Urology, Weill Cornell Medical College, New York Presbyterian Hospital, New York, NY;; ‡ University Teaching Hospital, Lusaka, Zambia;; § EngenderHealth, Nairobi, Kenya;; ‖ Impact Research and Development Organization, Kisumu, Kenya;; ¶ FHI 360, Durham, NC;; # Homa Bay District Hospital, Homa Bay, Kenya; and; ** ZPCT, FHI 360, Lusaka, Zambia.

**Keywords:** postcircumcision abstinence, ShangRing, circumcision devices, adult male circumcision

## Abstract

**Background:**

Men's understanding of counseling messages after voluntary medical male circumcision (VMMC) plays an important role in whether they follow them. Data on triggers for early resumption of sex may be useful as scale-up of VMMC for HIV prevention continues in sub-Saharan Africa.

**Methods:**

Data on understanding of post-VMMC abstinence recommendations, resumption of sex, condom use, and triggers for resuming sex were collected from participants during a follow-up interview 35–42 days after ShangRing circumcision in Kenya and Zambia.

**Results:**

Of 1149 men who had ShangRing circumcision, 1096 (95.4%) completed follow-up. Nearly all (99.2%) reported being counseled to abstain from sex post-VMMC; among those, most (92.2%) recalled the recommended abstinence period was 6 weeks. Most men (94.1%) reported that the counselor gave reasons for post-VMMC abstinence and recalled appropriate reasons. Few (13.4%) men reported resuming sex at 35–42 days' follow-up. Among those, 54.8% reported never using a condom post-VMMC. Younger participants (odds ratio 0.3, 95% confidence interval: 0.2 to 0.5, *P* < 0.0001) and those reporting at least some condom use at baseline (odds ratio 0.5, 95% confidence interval: 0.3 to 0.7, *P* = 0.0003) were less likely to report resuming sex. Among men who reported some condom use, most (71.5%) said condoms were much easier or easier to use after circumcision. Men reported various reasons for early resumption of sex, primarily strong sexual desire (76.4%).

**Conclusions:**

Most men reported awareness of and adherence to the counseling recommendations for post-VMMC abstinence. A minority reported early resumption of sex, and, among those, condom use was low. Results could be used to improve post-VMMC counseling.

## INTRODUCTION

Voluntary medical male circumcision (VMMC) dramatically reduces risk of HIV acquisition in adult men.^1–3^ Since 2007, when the World Health Organization (WHO) and the Joint United Nations Program on HIV/AIDS (UNAIDS) recommended that VMMC be considered for HIV prevention in settings with high HIV prevalence and low rates of male circumcision,^4^ approximately 6 million circumcisions have been performed in 14 countries in sub-Saharan Africa.^5^ Although the original goal of performing nearly 21 million male circumcisions by 2016 is unlikely to be met, scale-up of VMMC has arguably been one of the most successful public health interventions in recent history.^5^

Modeling has shown that widespread scale-up of VMMC would lead to large reductions in HIV incidence in men and women and lead to billions of dollars of treatment costs averted.^6,7^ Indeed, scale-up of VMMC in Orange Farm, South Africa, seems to have led to a significant reduction in community levels of HIV.^3^

One advantage of VMMC over other available HIV prevention options is that it is a one-time procedure that does not require continuous action or behavior change to receive the HIV risk reduction benefits of the intervention itself.^5^ However, there are concerns about increased risk of HIV acquisition or transmission if men do not adhere to the recommended postcircumcision abstinence period while the wound heals^8,9^ or if they increase risky behaviors after being circumcised (ie, risk compensation).^10^ Either could decrease the overall impact of VMMC.

Although there is mounting evidence that risk compensation is not widespread after VMMC,^1,3,10–12^ issues surrounding early resumption of sex and its associated risks, if any, are not clear. The WHO recommends a 42-day abstinence period after circumcision to ensure adequate time for wound healing, to reduce chances of complications, and to reduce the risk of acquisition of HIV infection among recently circumcised HIV-negative men or the risk of HIV transmission to female partners of recently circumcised HIV-positive men.^4,13^ An increased risk of HIV acquisition or transmission has not been ruled out with resumption of sexual intercourse before complete wound healing.^8,14,15^

This article presents data on men's understanding of postcircumcision abstinence recommendations and on resumption of sex, condom use, and rationale for resuming sex during the recommended abstinence period.

## METHODS

### Study Setting, Design, and Participants

Data presented here are from a prospective study of ShangRing VMMC conducted during routine service delivery at 10 sites, 7 in Homa Bay County, Kenya and 3 in Lusaka, Zambia. The primary objective of the study was to estimate the rate of circumcision-related adverse events after ShangRing circumcision in routine service settings. Details of the study methods and data on occurrence of adverse events, course of wound healing, and acceptability of the device have been previously published.^16^

### Procedures

Briefly, healthy uncircumcised men 18–54 years old, seeking circumcision and who provided written informed consent, were circumcised with the ShangRing device as previously described.^17–19^ Both HIV-negative and HIV-positive men were enrolled in the study. Rings were removed 7 days after circumcision, and participants were asked to return for 1 additional follow-up visit 35–42 days after circumcision. HIV prevention and risk reduction counseling, including the WHO postcircumcision abstinence recommendations, was provided during the initial circumcision visit and all scheduled and unscheduled follow-up visits. At both the 7-day and 35–42-day follow-up, a genital examination was conducted and participants were interviewed.

Participants were interviewed at the final follow-up scheduled for 35–42 days after circumcision. Men who did not return to the clinic for the final follow-up were telephoned in an effort to encourage them to return to the clinic. When men still did not return, we conducted home visits where possible and as a last resort contacted participants by telephone. Data included here are from men who were interviewed at the 35–42-day follow-up at the study site, during a home visit, or over the telephone. The denominators for some variables differ because of missing data from some participants.

We conducted univariate logistic regression analysis to explore if age at VMMC (18–24 vs. 25 years and older), condom use during 6 months before VMMC reported at baseline (at least some condom use vs. never), and number of sexual partners in the 6 months before VMMC reported at baseline (2 or more vs. 1 or 2) related to resumption of sex before the 35–42-day follow-up visit. These analyses included only men who reported they were sexually active during the year before circumcision.

### Ethical and Regulatory Review

Ethical and regulatory approvals were obtained from FHI 360, the Kenya Medical Research Institute, the University of Zambia, the Kenya Pharmacy and Poisons Board, and the Zambian Pharmaceutical Regulatory Authority.

## RESULTS

A total of 1149 men received ShangRing circumcision between February and May 2012, 554 in Kenya and 595 in Zambia. Of note, 1096 men (95.4%) completed the study with most (1034/1096, 94.3%) being interviewed at the study site and the remaining being interviewed during a home visit (25/1096, 2.3%) or over the telephone (37/1096, 3.4%).

Baseline characteristics of men included in this analysis were similar to those of all men in the study (Table 1).^16^ Participants in Kenya were younger than those in Zambia; 63.3% (335 of 529) of Kenyan participants were 18–20 years old compared with 22.8% (129 of 567) of those in Zambia. Approximately 75% of participants who completed the study reported at baseline that they had sexual intercourse in the past year, with most reporting 1 partner in the past 6 months. Reported current condom use at baseline was variable (Table 1).

**TABLE 1.**
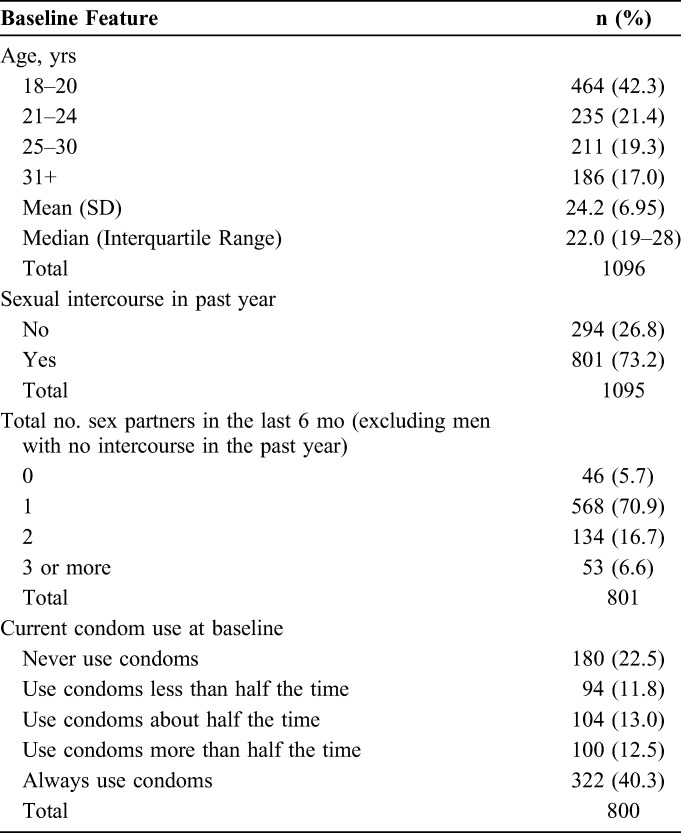
Selected Baseline Characteristics of Men Returning for Follow-up 35–42 Days After ShangRing Circumcision in Kenya and Zambia

Nearly all men (1073/1082, 99.2%) reported being counseled to abstain from sexual activity, including masturbation, after circumcision. Among those, most (981/1064, 92.2%) recalled that the recommended period was 6 weeks. A few recalled time periods less than or more than 6 weeks, stated “when the wound is healed,” did not remember, or said that the counselor did not specify.

Most men (991/1053, 94.1%) reported that the counselor gave reasons for abstaining postcircumcision; among them, all (990/990, 100%) reported one or more of the following reasons that abstinence is optimal (individually or in combination): allows the wound to heal (712/990, 71.9%), reduces the risk of or prevents disease/infection (231/990, 23.3%), or avoids injury, complication, wound disruption, or bleeding (229/990, 23.1%).

Few (143/1070, 13.4%) men reported resuming sexual activity before their 35-42 day interview. The majority (112/143, 78.3%) of those who reported resuming sex did so between 28 and 42 days after VMMC (Table 2). Among all men returning for follow-up, more men in Zambia reported resuming sex before 42 days than in Kenya, 13.2% (75 of 567) vs. 8.3% (44 of 529), respectively. Some final follow-up interviews were conducted after 42 days of circumcision, and, among those men, 22 reported resuming sex after the 42-day abstinence period (Table 2). Thus, overall, 11.3% (121 of 1070) of participants resumed sex early relative to the WHO recommendations. Participants who were 18–24 years old [odds ratio (OR) 0.3, 95% confidence interval (CI): 0.2 to 0.5, *P* < 0.0001] and those who reported at least some condom use at baseline (OR 0.5, 95% CI: 0.3 to 0.7, *P* = 0.0003) were significantly less likely to report resuming sex at their 35–42 day interview. Number of sexual partners in the 6 months preceding VMMC reported at baseline was not associated with resumption of sex at final follow-up (2 or more vs. 0 or 1 partner: OR 1.3, 95% CI: 0.8 to 2.0, *P* = 0.29).

**TABLE 2.**
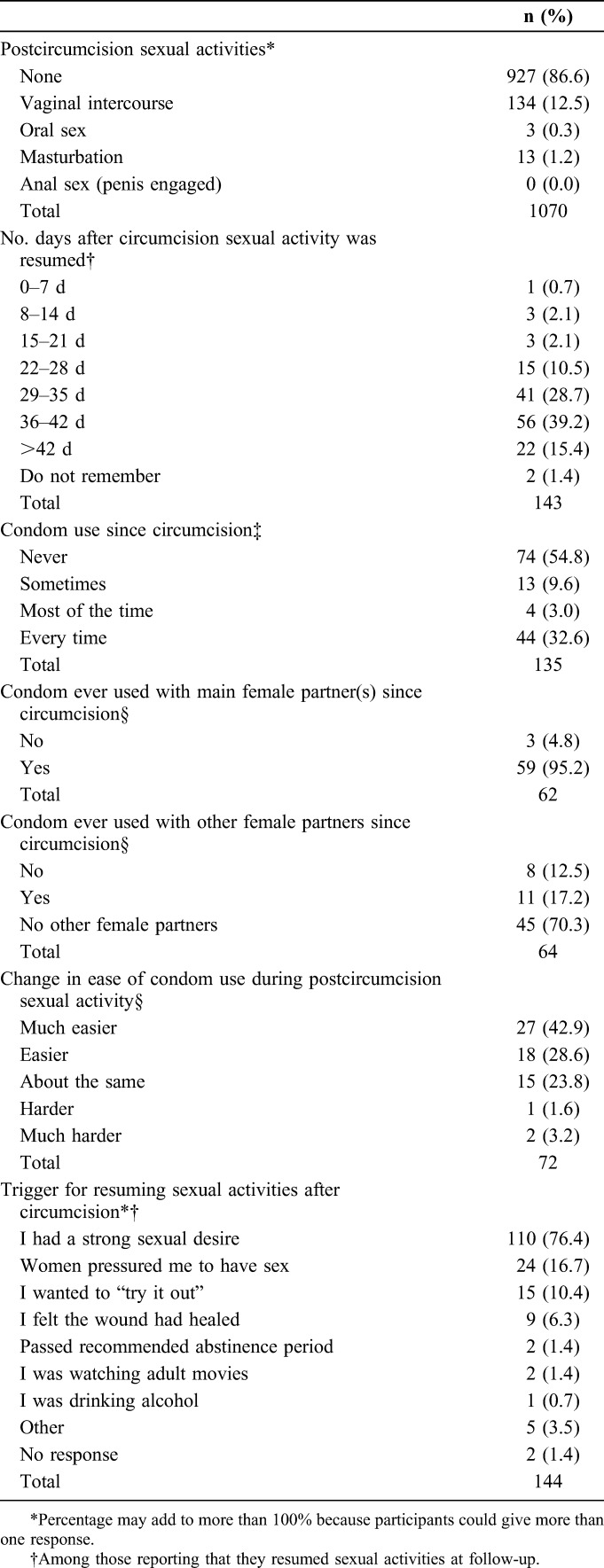

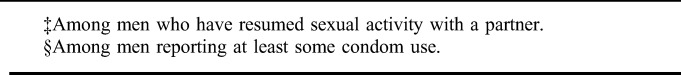
Reported Sexual Activity at Follow-up Among Men Circumcised With the ShangRing

Among those who reported resuming sex at follow-up, most (130/143, 90.9%) reported having only 1 sex partner since circumcision. The majority (87/143, 60.8%) of men who reported resuming sex said that sex was more pleasurable after circumcision, with one-third (48/143, 33.6%) saying there was no change and few (8/143, 5.6%) saying it was less pleasurable than before circumcision.

Among men who reported resuming sex with a partner, condom use was variable, with over half (74/135, 54.8%) reporting that they had not used a condom since circumcision (Table 2). Among participants reporting at least some condom use, most reported using a condom with their main sex partner; condom use with other female partners was less common. Among men who reported some condom use, the majority (45/63, 71.4%) said that condoms were much easier or easier to use after circumcision (Table 2). Most (129/138, 93.5%) men who reported they engaged in sex before the final follow-up interview said they had told their female partner(s) about the need for postcircumcision abstinence and, for the most part, their partner reacted positively (103/129, 79.8%).

When asked an open-ended question about why they resumed sex, strong sexual desire was the most common reason, cited by 16.7% (24 of 144) of participants (Table 2). However, when specifically asked if they felt pressured by their female partner(s), the percentage rose to one-quarter (34/135, 25.2%). Most (32/34, 94.1%) men said they felt pressured to have sex because their partner expressed a desire to have sex or her actions were suggestive. Other ways they felt pressured included that their partner thought the participant was cheating on her/asked why he was avoiding her (5/34, 14.7%), that she made comments that he was not a man if they did not have sex (3/34, 8.8%), or that she told him if they did not have sex he did not love her (2/34, 5.9%).

## DISCUSSION

Men in this study had good recall and understanding of postcircumcision abstinence counseling when interviewed approximately 6 weeks after VMMC. Nearly all remembered the counselor told them about abstaining from sex after VMMC; most were aware that the recommended abstinence period was 6 weeks and stated appropriate reasons for the need to abstain. Other studies in the area where our Kenyan sites were located reported that communication activities were successfully educating men and women about VMMC and the partial protection it provides.^20–22^

The 42-day abstinence recommendation is supported by results of studies showing that most men were healed^23,24^ and that there was no detectable penile HIV viral shedding in most men by 42 days after conventional VMMC.^15^ Approximately 11% of participants in our study reported resuming sex before the WHO-recommended 42-day abstinence period, with rates reported by others varying from approximately 4% to nearly 40%, perhaps in part due to different study methods.^8,23,25–29^ Rates toward the higher end are more likely to reflect those seen in typical services vs. study settings, where follow-up is less frequent, risk reduction counseling more limited, and there is a broader socio-demographic profile among those undergoing VMMC than in the studies where lowest levels of early resumption of sex have been reported.^8,23^

Resuming sex before 42 days does not necessarily mean men are resuming sex before complete wound healing. Odoyo-June et al^26^ found that taking into account results of a clinical assessment for wound healing and reported condom use, only approximately 7% of men had unprotected sex before complete healing after conventional VMMC. Most participants who reported resuming sex in our study said they did so more than 28 days after VMMC, similar to what has been reported elsewhere.^23,25^ Presumably, there is less HIV transmission risk when men resume sex toward the latter end of the abstinence period than earlier on. However, given the slower wound healing that has been seen after circumcision with devices in adults,^29–34^ these men could be at risk of HIV for longer during the healing period relative to conventional surgery. This requires further assessment to decide if the WHO recommendation should be revised in the context of device-based circumcision.^31^

We found that strong sexual desire was the primary reason for resuming sex before final follow-up. Additionally, one-quarter of men reported they felt pressure from their partner(s) to have sex. In a recent study in the same region where our Kenyan sites were located, men who mentioned the abstinence period as a possible barrier to VMMC thought it would be difficult to suppress the urge to have sex and that their partner might be unhappy about the need to abstain.^35^ Those men who said the abstinence period was not a barrier thought that if their partner understood the importance of abstaining, it would not be an issue.^35^ Most participants in our study said they told their female partners about the abstinence period and that their reactions were positive. Taken together, these results suggest that while women may generally agree with the abstinence recommendation, they may find it difficult to abstain for the full 6 weeks, as do the men themselves.

We found that men who were 25 years of age and older were significantly more likely to resume sex before the final follow-up interview. Others have reported that married men or those living with a sexual partner are more likely to resume sex early.^23,25–27^ We did not have data on marital status, although it seems plausible that the men in our study who were 25 years and older were more likely to be married than the younger participants. One advantage of circumcising younger men and adolescents is that they are less likely to have established sexual partnerships, be sexually experienced, and resume sex early after VMMC.^23,25,26^ For most of them, except for abstaining from masturbation, abstinence is not an issue. This has programmatic implications for promotion of VMMC.

Men in our study who reported at baseline that they had not used condoms in the 6 months before VMMC were significantly more likely to resume sex before the final follow-up interview. Others have found that men who report risky behaviors before VMMC are more likely to resume sex during the recommended abstinence period.^25–27^

Reported condom use was low among men in our study who resumed sex before the final follow-up, although most men who used a condom after VMMC reported that they were easier or much easier to use after circumcision than before, confirming findings by others.^12^ We saw large differences in postcircumcision condom use between the sites in Kenya and Zambia. Our results from Kenya mirror those reported by others in Western Kenya.^23^ Hewett et al^27^ found that risky behaviors including unprotected sex and multiple partners were not uncommon during the abstinence period.

Some limitations should be kept in mind. Data were from men 18 years or older who participated in the study and were interviewed approximately 6 weeks post-VMMC. They may not be representative of all men seeking VMMC. However, given that the study was designed to follow normal provision of services and that the proportion of men included in this analysis was high, and with similar baseline characteristics to the entire study group, the results likely represent men seeking VMMC in the geographical areas where the sites were located.

The study was not specifically designed to look at the issues presented here, and data on some covariates of interest were unavailable. Given that there was only 1 scheduled visit after ShangRing removal, it was not possible to determine the status of wound healing at the time participants reported resuming sex. Data collected during interviews were self-reported and thus subject to inherent inaccuracies and reporting biases such as recall or social desirability bias. For some variables, we were missing data from some participants. We cannot exclude this as a potential source of bias; we assume, however, that such data were missing at random.

As VMMC continues to be scaled up in sub-Saharan Africa, programs need to strengthen counseling to reduce risks during the postcircumcision abstinence period. Qualitative data on which benefits of post-VMMC abstinence are most compelling or how men and women could manage sexual desire during the abstinence period could lead to development of useful counseling messages. Information campaigns and counseling messages highlighting that many men find condoms easier to use and sex more pleasurable after circumcision, may reassure prospective clients and encourage condom use after VMMC. Couples counseling before circumcision or other ways to involve female partners or women more generally in VMMC could be explored as one possible way to reduce sex during the abstinence period.^25^ Other reasons to engage female partners in VMMC education and/or counseling are to improve their understanding of the partial protection VMMC provides and also because in some situations women are playing an important role in encouraging men to seek VMMC.^21,36,37^ Finally, it may be useful to explore use of targeted counseling approaches directed at men who report risky sexual behaviors before circumcision to reduce early resumption of sex after VMMC.
